# Species-Specific Spatial Patterns of Variation in Sexual Dimorphism by Two Lizards Settled in the Same Geographic Context

**DOI:** 10.3390/ani13040736

**Published:** 2023-02-18

**Authors:** Roberto Sacchi, Marco Mangiacotti, Stefano Scali, Federico Storniolo, Marco A. L. Zuffi

**Affiliations:** 1 Department of Earth and Environmental Sciences, University of Pavia, I-27100 Pavia, Italy; 2 Museo di Storia Naturale, Comune di Milano, I-20121 Milano, Italy; 3 Natural History Museum, University of Pisa, I-56011 Calci, Italy

**Keywords:** sexual dimorphism, geometric morphometric, islands, common wall lizard, Italian wall lizard

## Abstract

**Simple Summary:**

Understanding how sexual dimorphism responds to natural and sexual selection is essential to figuring out how intraspecific phenotypic diversity is produced. By comparing the response of two species of lizards inhabiting the same archipelago, we show that sexual dimorphism is a complex phenomenon resulting from the interaction between sexual and natural selection. These two forces act simultaneously, not necessarily in the same direction, and may generate species-specific spatial pattern of morphological variability even in species settled in the same geographic context.

**Abstract:**

The evolution of sexual dimorphism (SD) results from intricate interactions between sexual and natural selections. Sexually selected traits are expected to depend on individual condition, while natural selected traits should not be. Islands offer an ideal context to test how these drivers interact with one another, as the size is a reliable proxy for resource availability. Here, we analysed SD in body size (snout-vent length) and head shape (assessed by geometric morphometric) in two species of lizards (*Podarcis muralis* and *P. siculus*) inhabiting the Tuscan archipelago (Central Italy). We found a strong SD variation among islands in both species. Furthermore, in *P. muralis* emerged some significant correlations between SD and island size, supporting the occurrence of possible effects of individual condition on SD. By contrast, SD in *P. siculus* followed opposite trajectories than in *P. muralis*, suggesting that in this species, natural selection could play a major role as a driver of SD. Our findings show that natural and sexual selection can interact in complex ways, and the responses are species-specific. Therefore, spatial patterns of variation in SD may strongly differ among species, even when they settle in the same geographic contest.

## 1. Introduction

Sexual dimorphism is a widespread phenomenon in animals, and depends on a complex interaction among a variety of ecological, physiological and behavioural traits affecting reproduction [1,2]. Since Darwin [3], two main factors have been recognized to drive sexual dimorphism, namely, sexual selection, which accounts for the interactions between sexes, and natural selection, which deals with the interaction between animals and the environment [4]. The two processes are not mutually exclusive and can interact, either positively or negatively, to produce the observed intensity of sexual dimorphism [5,6]. However, the way natural and sexual selection processes work to generate dimorphism is substantially different, leading to predictable, or not, outcomes. Sexual selection promotes dimorphism mainly through male secondary sexual traits, which affect the male ability to obtain mates via intrasexual or intersexual competition [7,8]. Consequently, the expression of those traits associated with courtship or combat will be biased in males and not in females. On the other hand, natural selection promotes dimorphism through traits affecting the amount of resources individuals can intake and allocate to reproduction [4]. Typically, female-biased sexual dimorphism evolves in traits associated with fecundity, such as body size and any other trait that facilitates energy allocation to eggs production [2,4,9]. However, under certain ecological pressures, sexual dimorphism can evolve even without a direct link to reproduction [1]. For example, sexes may diverge towards a trophic structure in order to reduce the overlap in diet (competition between sexes), or when there are alternative ecological optima and each sex evolves towards a different one [1].

Traits involved in sexual dimorphism generally depend on the individual condition [7], and, consequently, show high intraspecific phenotypic plasticity according to spatial gradients of resource availability [10]. Typically, theory predicts that dimorphism in secondary sexual traits is strongly dependent on individual condition (condition-dependent sexual dimorphism) [7]. Hence, differences between sexes are locally much stronger when individuals (males) are in better condition, and males’ traits are more strongly affected by variation in conditions than females [7]. Several examples have been reported in different traits and species, particularly in insects [7,11,12,13,14], but also in vertebrates, even if mainly at a correlational level [15,16,17].

Ecological causes of sexual dimorphism rely on intrinsic differences in niches between males and females, or intersexual competition for limiting resources, which both interact with, and depend on, environmental conditions [1]. For example, sexual dimorphism in *Anolis* lizards on islands is reduced in the presence of potentially competing species [18]. Since the environment strongly affects the individual condition as well as the expression of fitness-related traits, even sexual dimorphism controlled by natural selection is expected to show condition-dependent expression and geographic variation depending on environmental spatial gradients [10,15].

Lizards are an ideal model for testing hypotheses on the evolution and maintenance of sexual dimorphism, since they show strong variation in sexual dimorphism in several traits within and among species [19]. Namely, Lacertid lizards show male-biased sexual dimorphism, with males having larger body size and larger heads affecting the ability to win intrasexual competition [20,21]. On the other hand, trunk length is known to be under fecundity selection in female lizards, as it correlates with the space available for egg storage and, consequently, it may affect the quality and quantity of the progeny [21,22]. Furthermore, head shape is also highly variable among lizards, and it relies on feeding, refuge and habitat use, as well as on competitive interactions and mating in males, being potentially under the influence of both natural and sexual selection [23,24,25,26]. Finally, lizards commonly inhabit insular systems, which offer good opportunities to analyse how condition-dependent traits are expressed in several populations of the same species under different environmental regimes [10,15].

**Figure 1:**
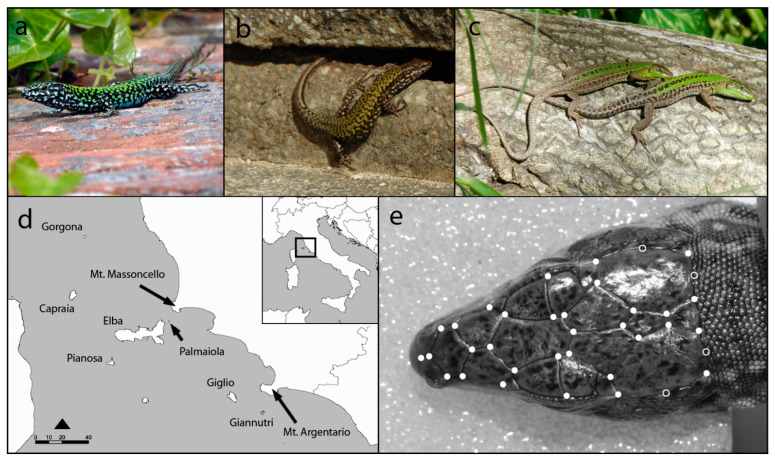
Species involved in the study: common wall lizard (*Podarcis muralis*) (**a**) male and (**b**) female; (**c**) Italian wall lizard (*Podarcis siculus*) male (lower individual) and female (upper individual); (**d**) islands and paleo-islands of the Tuscan Archipelago inhabited by species; (**e**) location of landmarks (filled circles) and semi-landmarks (open circles) used to analyse head shape of lizards on a *P. muralis* male.

In this study, we analysed the relationship between sexual dimorphism in head shape and island size in two species of lizards (the common wall lizard, *Podarcis muralis*, and the Italian wall lizard, *Podarcis siculus*) inhabiting the same archipelago (the Tuscan archipelago). *P. muralis* (Figure 1a,b) is a medium-sized lacertid, occurring in many south-western European countries [27], which shows a high ecological plasticity which allows the colonization of many different habitats, from urban areas to natural environments, including cultivated areas. *P. siculus* ( Figure 1c) is a small diurnal lizard endemic to the Italian peninsula [27] occurring in a high variety of habitats, but it prefers open habitats, with tall vegetation and with high levels of insolation [28]. In both species, males are larger than females and have a larger head compared to body size [28,29]. The trophic ecology of both species has been extensively studied [28,29,30,31,32,33], and all analyses described them as generalist predators. 

The Tuscan archipelago (Figure 1d) consists of a group of seven major islands, of which the largest is the island of Elba, plus some minor, dry and rocks located between the Tuscan mainland and Corsica. The islands are characterized by the presence of numerous endemic species (especially birds and reptiles), while mammals are those typical of the Mediterranean environment. Potential predators for lizards are the whipsnake *Hierophis viridiflavus*, present in all the studied islands, and the Smooth snake, *Coronella austriaca*, present on the Elba island [34], while competitors are virtually absent or not known (MALZ unpubl. data)

Assuming that the size of the island is a proxy of the resource availability, we specifically assess i) if the sexual dimorphism increases in larger compared to smaller islands, ii) if species respond similarly to the island size, and iii) if the variation in head-shape dimorphism is more pronounced in one sex according to the prediction of condition-dependent sexual/natural selection.

## 2. Materials and Methods

We analysed 180 adult specimens (Table 1) preserved in the “La Specola” Zoological Museum (Florence, Italy), including 118 *P. muralis* (69 males and 49 females) and 62 *P. siculus* (35 males and 27 females). Specimens were collected between 1952 and 1999 and covered all the 10 islands of the Tuscan Archipelago and two paleo-islands along the coastline (Mount Argentario and Mount Massoncello [35], Figure 1d). The snout-vent length (SVL) of each individual was measured (to the nearest mm) with a digital calliper, and island size (ha) was obtained from Bonardi et al. [36]. 

For each lizard, we obtained a head image using a Nikon D50 camera at a 1.2 million pixel resolution, equipped with a Nikkor 60 mm AF-S Micro lens, at a fixed distance of 18 cm. Head size and shape variables were obtained using landmark-based geometric morphometrics. Head geometric morphometrics was performed using 32 landmarks and four semi-landmarks located at intersections and borders of cephalic scales (Sacchi et al., 2015, Figure 1b). A TPS file with all the specimens of the two species was created and landmarks were digitized using tpsUtil and tpsDig2 [37]. Specimens were scaled to unit centroid size and superimposed using a generalized Procrustes analyses (GPA). For each specimen, we computed a new perfectly symmetric landmark configuration [15,38].

Firstly, we fitted a linear model to check if sexual dimorphism in body size differed between species and depending on island size. The SVL was the dependent variable, whereas the sex, species and island size (ha) were the main effects. We also added the three-way interaction between the main effects to account for possible species-specific allometric effects of sexual dimorphism on body size. 

In order to analyse how sexual dimorphism in head shape varies depending on island size and between species, we fitted a linear model with a randomized residual permutation [39]. The model decomposes the distance matrix of specimens’ coordinates according to the fixed effects and checks for statistically significant effects through a randomized residual permutation procedure. Fixed effects were the species, sex, specimens’ size (the logarithm of the centroid size; henceforth, lnCS), and island size. These variables entered the model as a four-way interaction, in order to look for condition-dependent sexual dimorphism (sex × island size interaction) and for possible effect of body size (sex × island size × body size interaction) and species-specific trends (sex × island size × body size × species interaction). 

We used trajectory analyses [40] to analyse the outcome of the model in order to assess if the magnitude of vectors of sexual dimorphism was greater in large than in small islands, and if the directions (i.e., the angles between the vectors of sexual dimorphism) diverged or not between species. The same analysis was used to investigate the phenotypic changes from small to large islands within sex in order to check if males vary more than females. 

A principal components analysis (PCA) was carried on the variance–covariance matrix of the landmark coordinates, and the first two components were used to represent the phenotypic trajectories predicted by the model for both sexual dimorphism in small and large islands and phenotypic changes in males and females in both species [40]. Significant differences in both magnitude and direction between phenotypic vectors were assessed through permutations (n = 999) of model’s fitted values [40]. Finally, patterns of shape variation were visualized using thin-plate spline (TPS) deformation grids.

The linear model and TPS grids were obtained using the *RRPP* [41] and *geomorph* [42] packages, respectively, in R ver. 4.2.1 [43]; unless otherwise stated, data reported are means and standard errors.

## 3. Results

**Figure 2:**
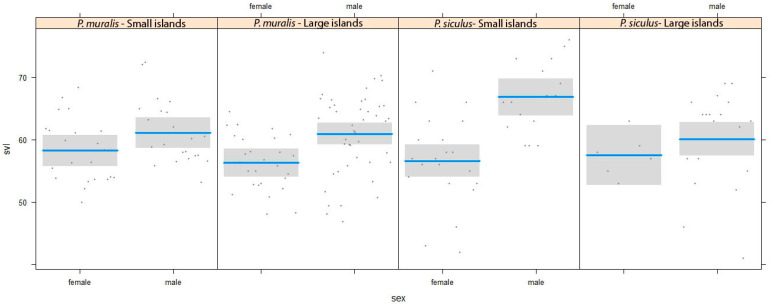
Conditional plot showing the relationship between sexual size dimorphism and island size for *P. muralis* and *P. siculus*. Female–male differences are divided into two categories of island size: small islands (lower than median) and large island (higher than median).

The linear model for sexual dimorphism in body size (SVL) showed that, in both species, males were larger than females (F_1,172_ = 25.94, P < 0.001, Figure 2), and no effect of island size was appreciable (the sex × island size interaction was not significant: F_1,172_ = 1.260, P = 0.26). However, in a second analysis in which island size was divided into two categories of size (small and large according to the median value, Table 1), a significant species-specific effect of the island emerged (sex × island size × species interaction: F_1,172_ = 5.112, P = 0.024). the sexual dimorphism of *P. siculus* was more evident in small than in large islands, whereas no difference was detected in *P. muralis* (Figure 2).

The linear model for the head shape (Table 2) revealed a significant four-way interaction, suggesting that sexual dimorphism is species-specific and varies in a complex way according to island and body size (F_1,164_, P = 0.035). At the same time, we found a highly significant sex × area × species interaction (F_1,164_, P = 0.004), suggesting that sexual dimorphism depends on the island size, but with species-specific patterns. Finally, the interaction between island size and lnCS was also significant (F_1,164_ = 3.073, P = 0.002), supporting the fact that island size also affects the allometric pattern in addition to the sexual dimorphism.

**Figure 3:**
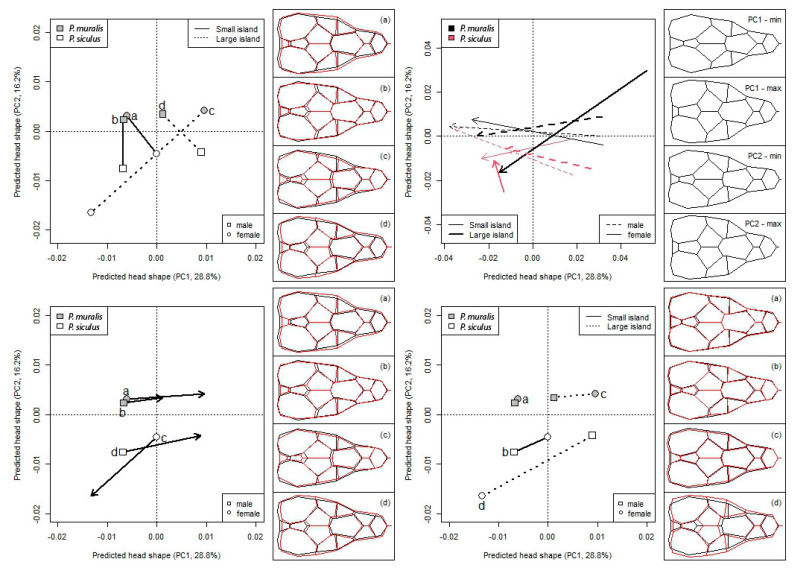
Phenotypic vectors for difference in head morphology between species according to sex, head and island size with the mean landmark configuration in the right panels. Top left: species differences within sex; panels report *P. muralis* in black and *P. siculus* in red. Top right: allometric trajectories; large and small islands as in Figure 1. Bottom left: phenotypic displacement of sexes in each species from small to large islands; panels report small islands in black and large islands in red. Bottom right: species sexual dimorphism in small and large islands; panels report males in black and females in red.

The trajectory analysis on the PC scores computed on the model’s fitted values revealed that the primary pattern of variation in head shape depends on species (Figure 3) and shows a strong allometric response (Figure 3). The phenotypic difference vectors between species within sex differed in magnitude (P < 0.011), but not in direction (P > 0.602). *P. siculus* had more stubby heads (wider and shorter) than wall lizards and the difference between species was more evident in larger than smaller islands, especially for females (Figure 3). Indeed, the magnitude of the species’ phenotypic vectors did not significantly differ between sexes in small islands (females: 0.0165, males: 0.0174, P = 0.789), whereas the difference between the females was almost double that of the males in the larger islands (females: 0.0335, males: 0.0198, P_dif>0_ < 0.001).

Both species showed a similar ontogenetic pattern (angle among vectors did not differ from zero for any pairwise comparison: P > 0.537), the head becoming gradually slender and pointed as the size increases (Figure 3). However, the allometric change was significantly larger than zero in both sexes of *P. muralis* (males: P < 0.022; females: P < 0.006), whereas it was only for males in *P. siculus* (small islands: P = 0.022; large islands: P = 0.096), but not for females (P > 0.143). Consequently, the magnitude of the allometric change did not differ among species and sexes in small islands (Figure 3), while *P. muralis* females displayed a significantly wider allometric change than both male and female *P. siculus* (P < 0.015, Figure 3) in large islands.

The phenotypic displacement in head shape from small to large island (Figure 3) was significantly higher than zero in all cases (P < 0.008) and did not differ among species and sexes (P > 0.232). Furthermore, the phenotypic vectors were oriented in the same direction (angles did not significantly differ from 0° in any pairwise comparison: P > 0.700), but *P. siculus* females were opposite-oriented with respect to both conspecific males and all *P. muralis*. 

Finally, sexual dimorphism in both species was larger in large than in small islands (Figure 3). Indeed, phenotypic vectors did not significantly differ from zero (*P. muralis*: P = 0.147; *P. siculus*: P = 0.067), and between species (P = 0.348) in small islands. By contrast, in large islands, the phenotypic vector for sexual dimorphism differed from zero in both species (*P. muralis*: P = 0.023; *P. siculus*: P < 0.001), and was higher in *P. siculus* than in *P. muralis* (P = 0.006). All phenotypic vectors were oriented in the same direction (all pairwise comparison being not significant: P > 0.194). Since the trajectories of the phenotypic change in the females of the two species are opposite (Figure 3), the phenotypic change in sexual dimorphism from small to large island is also opposite between species (Figure 3, right panels). Males’ heads are more slender and elongated than females’ in *P. muralis*, while they are more stocky and wider in *P. siculus*.

## 4. Discussion

Understanding how sexual dimorphism responds to natural and sexual selection is essential to figuring out the general processes causing both intra- and interspecific phenotypic diversity. By comparing the response of two species inhabiting the same insular system, we suggest that the sexual dimorphism of *P. muralis* and *P. siculus* may diversify under similar, but not identical, processes. Indeed, we clearly showed that sexual dimorphism responded to island size in both species, but the patterns of response were species-specific. In summary, island-specific effects emerged especially for head shape, whereas they were less evident for body size. Irrespective of island size, males had a larger body than females, and the island effect occurred only in *P. siculus*. However, the pattern of response in this species was contrary to expectations, since size dimorphism was more intense in small rather than in large islands. On the other hand, in both species, the sexual dimorphism of head shape was larger in large islands, but with opposite general patterns in the two species. First, the two sexes differed mainly in the shape of the area around the back of the head, and males had longer parietal scales compared to females in *P. siculus*, while the reverse was true for *P. muralis*. Interestingly, these patterns basically replicated those observed in two previous studies, which, however, had been performed at the species level and not from a comparative perspective such as that adopted in the present study [10,15]. Second, the two species also differed in how the larger sexual dimorphism was achieved as the size of the island increases. Indeed, males and females of each species followed their own phenotypic trajectories. In *P. muralis*, both sexes followed the same phenotypic trajectory with a similar displacement from small to large islands, whereas in *P. siculus*, males and females followed divergent trajectories, with females having a longer displacement than males. Interestingly, this divergence between sexes agreed with data reported for *P. siculus* in the Eolian archipelago, where opposite trajectories of phenotypic change also emerged in both body size and head shape [10]. We acknowledge that sample size was not always optimal [44], but we are confident about reliability of general results since the effects of low sample size is normally to increase variance and, consequently, mask relationships. 

Several studies have previously demonstrated variation in sex-related traits with island features, specifically because islands offer different contexts of resource availability and environmental features [10,45]. When affected by individual condition, sexual dimorphism is expected to increase as resource availability grows [2,9]. Furthermore, if traits are also targeted by sexual selection, males are expected to respond more readily than females to the increased resource availability, since the expression of secondary sexual characters entails high energetic costs [7]. The study carried out on the Eolian islands supported the occurrence of a certain degree of condition dependence in the sexual dimorphism of *P. siculus*, especially in body size [10]. In fact, a positive correlation between body condition and the expression of sexual dimorphism in both body size and head shape was actually found, although it was much less evident on head shape [10]. Our study was based on museum specimens, so we were not able to retrieve any measure of individual body condition. Therefore, we used the island size as a proxy for it. Even though no direct measurement of the island features potentially affecting the individual condition of lizards was performed, several studies have shown that the size of an island can be used as a reliable proxy for resource availability. This is because larger islands offer more resources than small ones and populations in large islands experience less demographic stochasticity [15,46]. Specifically for *P. siculus*, a strict collinearity between size and ecosystem productivity actually emerged in the Eolian archipelago [10]. Therefore, the finding that sexual dimorphism, especially in head shape, increased with island size is consistent with the hypothesis that it might be correlated with individual condition. However, for the Tuscan Archipelago, these considerations better applied to *P. muralis* than to *P. siculus*. In the first species, as the size of the island grows, the sexual dimorphism increases in terms of body size (even though, not significantly, males are always larger than females), and in terms of head shape. This confirms previous findings [15], and fits the predictions from the hypothesis that sexual selection can contribute to promoting variability in sexual dimorphism among populations (i.e., islands) through condition-dependent expression of traits involved in sexual competition (namely, male-male combats). Accordingly, the difference in head shape between males and females relates to the parietal scales, and males show a relatively larger head than females, especially in larger islands. The enlargement of the heads in the tympanic area in males of the *Podarcis* lizards has been related with bite performance [26]; therefore, in larger islands, males could be able to bite with increased force compared to males in small islands, consequently performing more efficiently in intra-sexual competition. 

It is more difficult to argue that the mechanism of the dependent condition is also sexual in *P. siculus*, since in this case females have relatively wider heads in larger islands; they show the largest phenotypic displacement, and the sexual dimorphism of body size increased as islands became smaller. All the above results suggest that *P. siculus* morphology relates to individual condition more in females than males. The mechanism linking individual condition with size and shape in females is natural selection for increased fecundity (i.e., fecundity selection, [4]). Accordingly, when female fecundity is constrained by energy intake (i.e., individual condition), any trait associated with acquisition and processing of energy is favoured [1]. Therefore, the increased resource availability in larger islands could promote a relatively larger head in females, allowing them to improve the ability to collect energy from larger prey, and eventually maximize their reproductive abilities. Further, females also compete between themselves, so larger heads may be useful for intrasexual competition. This cannot happen in small islands, leading to an increase in the difference in head shape between males and females. In this scenario, it would be natural selection that determines the viability in the sexual dimorphism of the species in relation to the size of the islands, through condition-dependent expression of traits involved in female fecundity. If so, egg and clutch sizes are expected to correlate with island size in the same way as sexual dimorphism.

A further noteworthy result was that both species showed allometric variation in head shape, which differed between sexes and in relation to the island size. The allometric trajectories oriented in the same direction as that of the displacement from small to large islands. In many species of lizards, sexual dimorphism in both size and shape arises from sexual differences in static allometry, wherein males keep higher allometric slopes than females [22,47]. Here, we found that the allometric trajectory pointed in the same direction in both species, and differences occur mainly in the magnitude of the shape displacement, which correlates with island size. Notably, in the small islands, no difference between species and sexes within species was observed, whereas in large ones, displacements were longer and females tended to have a larger displacement than males, especially in *P. muralis*. This correlation between allometric displacement and island size pointed to condition dependence in allometry, suggesting that individual condition may affect the magnitude of the allometric change. Therefore, the variability of sexual dimorphism among islands of different sizes might also reflect the differences in static allometry related to individual conditions.

The head in lizards is involved in multiple ecological tasks other than those strictly related to individual condition, such as male aggressiveness or female fecundity. Indeed, head traits fulfil feeding habits and refuge use [24,26,48], they associate with microhabitat [49], and they can also influence predation risk [26]. Consequently, sexual dimorphism in head shape may result from niche segregation or competitive displacement in response to high competition between sexes [1,2]. When sexual dimorphism is driven by ecological causes not related with reproduction, the direction of dimorphism (i.e.., male or female biased) is not fixed, and it depends on the interaction between the ecological conditions and species life-history traits [1]. Even in the absence of a correlation between island size and individual condition (mediated by resource availability), variability in sexual dimorphism among islands of different sizes could still arise if the intensity of the intersexual competition for resources or optimal microhabitats covaries with island size. Species-specific patterns of response might, therefore, evolve given the divergence in the ecological niches between *P. muralis* and *P. siculus* [28,29].

Finally, the evolutionary history of the *Podarcis* genus in the Mediterranean Basin was particularly complex, with extensive genetic exchange between lineages, leading to mosaic genomes with contributions from two or more parental taxa [50]. In particular, *P. muralis* is a “pure” species which belongs to the Iberian clade, whereas the *P. siculus* is a mosaic species that evolves through hybridization between the ancestors of the Iberian and Balkan clade [50]. The divergence of the two species dates to around 16 million years ago [50]. The difference in the pattern of head-shape variability between the two species, notably the different phenotypic trajectories, might be the results of the different evolutionary trajectories they have followed since the time of their appearance.

## 5. Conclusions

Together, our findings show that sexual dimorphism in lizard is a complex phenomenon resulting from the interaction between sexual and natural selection including condition-dependent trait expression and allometric effects. All the above processes act simultaneously, not necessarily in the same direction, with different equilibria in species settled in the same geographic context. These complex interactions can cause variation in sexual dimorphism both within and among lizard species.

## Figures and Tables

**Table 1:** Samples of males and females used to estimate sexual dimorphism for each species in every island population. Type shows the subdivision of the islands into two groups (large and small) according to the median of the island size.

Island	Size (Km^2^)	Type	Species	Males	Females
Mount Argentario	60.23	large	*P. muralis* *P. siculus*	7 5	7 1
Capraia Island	19.24	large	*P. siculus*	3	5
Elba Island	223	large	*P. muralis* *P. siculus*	29 14	15 5
Giannutri Island	2.39	small	*P. siculus*	7	5
Giglio Island	21.47	large	*P. siculus*	3	7
Gorgona Island	2.27	small	*P. muralis*	10	10
Palmaiola Islet	0.09	small	*P. muralis*	13	9
Pianosa Island	10.41	small	*P. muralis* *P. siculus*	13 3	12 4
Mount Massoncello	34.59	large	*P. muralis*	10	5

**Table 2:** Results from statistical analyses of head morphology.

Model Predictor	df	F	*p*
Species	1,164	9.097	0.001
Sex	1,164	0.646	0.741
Island size	1,164	5.184	0.001
lnCS	1,164	44.172	0.001
Species × Sex	1,164	0.498	0.919
Species × Island size	1,164	1.763	0.065
Sex × Island size	1,164	1.428	0.149
Species × lnCS	1,164	1.348	0.193
Sex × lnCS	1,164	1.056	0.355
Island size × lnCS	1,164	3.073	0.002
Species × Sex × Island size	1,164	2.574	0.004
Species × Sex × lnCS	1,164	0.549	0.882
Species × Island size × lnCS	1,164	1.090	0.338
Sex × Island size × lnCS	1,164	0.750	0.672
Species × Sex × Island size × lnCS	1,164	2.148	0.035

## Data Availability

https://zenodo.org/badge/DOI/10.5281/zenodo.7650211.svg, accessed on 6 January 2023.
